# Raising Helpful Children: Exploring Conflict Between Goals and Practices in Euro-Heritage Socialization of Helping

**DOI:** 10.3389/fpsyg.2021.722998

**Published:** 2021-12-10

**Authors:** Luc Fairchild

**Affiliations:** Department of Human Development and Family Studies, University of Wisconsin-Madison, Madison, WI, United States

**Keywords:** socialization, prosocial development, parenting, culture, family dynamics, instrumental helping, indigenous, toddler

## Abstract

Helping others benefits both helper and helpee and is the basis for societal structures that promote collective well-being. Many parents Using a White, European-heritage “Constellation of practices” (UWEC), recognize the importance of raising a child who helps others. Yet UWEC children seem to take initiative to help with household work less, and in ways that benefit others less, than other children globally. It is important for future researchers to explore the phenomenon of many UWEC parents using practices that work against their explicit goals, and suggestions are made for future work, including better integration of cross-cultural evidence in developmental psychological study design. Better integrating evidence and exploring this conflict further would greatly advance our understanding of the socialization of helping, and may elucidate how much change is possible and advisable regarding how best to raise children to think and act in other-oriented ways that are beneficial for all.

## Introduction

Many United States parents regard raising children who help others as important (Pew Research Center [PRC], 2014). Yet white, European-heritage children tend to help in less complex ways and with less initiative than other children globally (Whiting and Whiting, 1973, 1975; Ochs and Izquierdo, 2009; Ochs and Kremer-Sadlik, 2013; Alcalá et al., 2014; Coppens et al., 2014, 2016). Their parents use practices associated with limited children’s helping (e.g., restricting children’s opportunities to help). Further exploring the conflict between parents’ values to raise helpful children and their practices can enhance understanding of parent socialization, and how parents/society can help children grow up to help others. This review highlights the importance of building theory and research grounded in cultural differences (Rogoff, 2003; Keller, 2018), as this white, European-heritage parenting conflict has long been described in cross-cultural studies (Whiting and Whiting, 1973), yet is rarely explored in work with those samples.

### Naming Note

While naming white culture disrupts its invisibility as a norm/standard toward which all should strive, the social construct of race often does not encompass similar cultural practices (Rogoff, 2003); many white parents may not use the practices referenced; many BIPOC (Black, Indigenous, and People of Color) parents may. The acronym UWEC focuses on what people do – their “constellation of practices” (after Rogoff, 2003), and refers to people “Using a White, European-heritage Constellation of practices” (UWEC), regardless of race or origin.

Developmental science often focusses on universal processes, yet historical bias in regarding non-UWEC cultures as “other” and lesser mirrors a siloing of methods and findings of cultural studies from other work, as seen in the study of children’s helping. Helping caregivers around the house is one of toddlers’ first opportunities for helping, and is akin to Instrumental helping, helping another person complete their goal. In “mainstream” work within UWEC communities, it is not the convention to measure elements of socialization highlighted in cross-cultural work and relevant to a conflict between values and practice (e.g., when age-dependent socialization strategies were related to toddler instrumental helping, Pettygrove et al., 2013; Dahl, 2015; Hammond and Carpendale, 2015; Dahl et al., 2017).

Mainstream models of instrumental helping have limitations. They often rely on laboratory paradigms for which external validity is not directly tested (cf. Yarrow et al., 1976, lab and observational measures were not related), and for which observational data is lacking (Dahl, 2017, 2019). Longitudinal instrumental helping studies have been limited to UWEC cultures where children tend to help less and with resistance across development. While an early seminal study suggested no relationship between early instrumental helping and pre-adolescent prosocial behavior, unlike other kinds of helping (Eisenberg et al., 1999), questions regarding instrumental helping paradigms’ relevance to concurrent child behavior offset limited UWEC longitudinal findings.

Cross-cultural methods often emphasize observations of children’s everyday lives, assuming that patterns of interactions with people and contexts are intrinsic to development (Vygotsky, 1978); cross-sectional studies show growth in early to late instrumental-type helping within a cultural context that nurtures helping. Integrating across these areas of study to generate theory, determine research questions and methods, design studies, and interpret results holds great promise in better understanding and supporting the socialization of instrumental-type helping. Better integration could help answer long-unanswered questions about UWEC parent practices conflicting with goals. Relevant suggestions are presented.

## Why We Should Care: Individual to Community Repercussions

Family practices encouraging children’s helping likely benefit individual, family, and community. Intervention studies suggest that when UWEC children act to benefit others, children’s well-being improves in measurable ways: higher quality of life, self-esteem, persistence, gratitude; and better cognitive problem-solving (Battistich et al., 1989; Umino and Dammeyer, 2016; Padilla-Walker et al., 2020). There are also implications for family well-being if what many UWEC parents want for their children regarding household contributions conflicts with what parents do every day and how children respond. In those situations, parents may experience low parenting self-efficacy (PSE), defined as parents’ belief that they are able to influence children toward successful development (Ardelt and Eccles, 2001). Because many UWEC parents spend a significant amount of time doing chores (Ochs and Kremer-Sadlik, 2013), low PSE in this context likely contributes significantly to lower PSE overall, which is related to higher parenting stress and depression (Bugental et al., 1993; Crnic and Ross, 2017). Further, PSE has been related to observed parenting behaviors, (e.g., responsivity and sensitivity), as well as child outcomes, including attachment status and emotion and behavior problems (Williams et al., 1987; Bugental and Cortez, 1988; Donovan et al., 1990; Coleman and Karraker, 1998; Jones and Prinz, 2005; Rominov et al., 2016; Wittkowski et al., 2017). Evidence suggests parent, child and family well-being decreases if parents experience low PSE, which may result from a conflict between UWEC parent practices and values regarding children’s helping.

Low PSE is thought to be expressed by negative affect, and low persistence (Bandura, 1977; Bugental et al., 1993; Jones and Prinz, 2005). In home observations, UWEC parents often demanded 5- to 17-year-old children do household tasks, then gave in; commonly expressing frustration or anger (Ochs and Izquierdo, 2009; Ochs and Kremer-Sadlik, 2013, 2015). Researchers’ observations of parents often “undermin(ing their) own authority,” suggest low persistence (p.733, Ochs and Kremer-Sadlik, 2015). Overall, observations depict conflict/confusion vs. an intentional parenting strategy, and suggest low PSE.

Helpful children might inspire more maternal sensitivity, a way of interacting regarded as beneficial to UWEC parent, child and family. Maternal sensitivity has been coded when a mother “responds promptly and appropriately to child’s cues, … and supports and scaffolds the child’s interests,” (p. 1741; Ainsworth et al., 1974; Feldman and Masalha, 2010; Newton et al., 2016). In laboratory settings, instrumental helping was positively associated with maternal sensitivity and scaffolding (Hammond and Carpendale, 2015; Newton et al., 2016), and a bidirectional relationship was suggested (Newton et al., 2014).

Children who gain skills to help may use them to help people outside the family. In many Indigenous-heritage families, children’s extensive helping spans home and community settings (Rogoff, 2003; López et al., 2012; Alcalá et al., 2014; Coppens et al., 2014, 2016). If parents can adopt practices that support and enhance children’s ability to help others and the group, this likely benefits the individual child, the family, and the community.

## What We Know From Cultural Comparison; The Conflict in Using a White, European-Heritage Constellation of Parenting Practices

A commonality exists among great global variation: from about 4–6 years of age, children tend to readily contribute to household work; Indigenous-heritage families of the Americas provide a strong example (e.g., Rogoff et al., 1980; de Haan, 1999; Gaskins, 1999, 2020; Rogoff, 2003). Children commonly help out of their own initiative in complex ways as they develop, and parents tend to prioritize children’s autonomy in participating collectively to benefit the family and community (e.g., Rogoff et al., 1993, 2017; de Haan, 1999; Gaskins, 1999, 2020; Ochs and Izquierdo, 2009). Parents commonly believe children are motivated and able to help from infancy, so that encouraging autonomy naturally leads to being “acomedido/a,” anticipating and providing the needs of others/the group (Rogoff et al., 1993, 2017; Rogoff, 2003; López et al., 2012; Alcalá et al., 2014; Coppens et al., 2014, 2016; Coppens and Rogoff, 2017). That research suggests many Indigenous-heritage communities regard individual autonomy from early years as essential to helping, and children’s participation should not be forced or controlled, but supported. These parents often emphasize children’s sensitivity and situational awareness, especially regarding others’/group needs, and often nurture children’s agency from birth, including acting to benefit others (Mosier and Rogoff, 2003). Toddlers’ participation is often seen as important for the whole group, as well as an expression of belonging (Rogoff, 2003; Alcalá et al., 2014; Coppens et al., 2014, 2016). UWEC scholars working with predominantly UWEC families rarely consider such factors regarding the group’s larger goals; “the group” might be the family or community.

UWEC parents tend to focus on self-care tasks vs. tasks helping the whole family (Ochs and Izquierdo, 2009; Ochs and Kremer-Sadlik, 2013; Coppens et al., 2016). They often control and assign children’s contributions and from early on, separate children from household work (Morelli et al., 2003; Ochs and Izquierdo, 2009; Alcalá et al., 2014; Coppens et al., 2016; Coppens and Rogoff, 2017; Rogoff et al., 2017). Though UWEC parents want to raise helpful children, these practices contrast with practices that are associated with children helping from their own initiative throughout development.

Researchers have regarded UWEC children’s limited development of initiative to help as problematic and have endorsed change (Whiting and Whiting, 1973; Ochs and Kremer-Sadlik, 2013). Whiting and Whiting (1973, 1975) suggested that children’s early experiences helping others were limited, noting parents’ over-emphasis of egoistic values while simultaneously encouraging children’s dependence (Whiting, 1978).

## Some Questions to Be Asked

How differences in children’s helping emerge remains an open question. UWEC parents might not see toddlers’ early bids to participate as a valuable early form of helping, as many Indigenous-heritage mothers recognize (Rogoff et al., 1993; Coppens and Rogoff, 2017). UWEC parents are likely missing cues of toddlers’ helping interest. For example, Coppens et al. (2020) found that UWEC mothers of 2- to 3-year-olds often rephrased questions about their children “helping” into descriptions using other action verbs. Some UWEC mothers believed their 2- to 3-year-olds to be incapable of helping from their own initiative (Coppens and Rogoff, 2017), despite evidence that UWEC toddlers are highly motivated to help (Rheingold, 1982; Warneken and Tomasello, 2013) and in many communities, take the initiative (e.g., Rogoff et al., 1993; Gaskins, 1999, 2020; Forman, 2007). Parents might then avoid scaffolding toddlers’ helping attempts toward successful helping. Coppens and Rogoff’s (2017) examples of UWEC mothers redirecting toddlers to other activities, or doing work when toddlers were not present, fit with the idea that those mothers did not see toddlers’ interest in helping, or did not regard it as an opportunity for children to learn to help skillfully. A study examining “unhelpful” toddler helping presented anecdotal data suggesting some parents did not guide those attempts: “I tell him what a good job he is doing, and when he’s not looking, I redo it” (p. 3, Hammond and Brownell, 2018). In this example, a proud toddler is left without understanding what developmentally appropriate actions might actually be helpful. Similarly, Coppens and Rogoff (2017) describe mothers giving toddlers “fake work” or alternatively, redoing failed toddler helping attempts without guidance or correction.

These parenting practices likely accumulate unsuccessful or discouraged toddler helping attempts if those attempts continue to go unrecognized or misread, or as parents continue disregarding toddlers’ current and future potential to help, and leave toddlers’ bids without support. It would then be expected that children eventually initiate helping less. This might explain how low maternal sensitivity, mothers misreading toddlers’ intent and potential, over time leads to lower toddler-initiated instrumental helping. Future work should examine this explanation, and whether parents’ restricted perceptions of toddlers’ abilities and motivations are thereby reinforced.

Other questions might productively drive future work. Parents seem to value raising helpful children according to survey data and observations that UWEC parents often try to get children to help (Ochs and Izquierdo, 2009; Ochs and Kremer-Sadlik, 2013; Pew Research Center [PRC], 2014; Dahl, 2015). It is not clear whether other values might be of similar significance. For example, Whiting (1978) noted that UWEC children tend to seek help and attention often, leading to less other-orientation, yet those seeking tendencies could be advantageous for achievement in schooling and employment. Achievement is often emphasized in middle-class, UWEC families even when children are toddlers (Kuserow, 2004). Future work should determine the relative importance of child tendencies UWEC parents want to nurture. The relative importance of many other UWEC values, practices and structural factors identified by researchers should also be explored.

Culture has been portrayed as invisible to participants, like water to a fish (Kluckhohn, 1949; Rogoff, 2003). Parents may need to “see” and change default practices to align their daily actions with values to raise helpful children. Accounts by Ochs and Kremer-Sadlik (2013, 2015) present many examples of stressed UWEC parents rushing to get things done. This state of mind may promote focusing on immediate needs and tasks in habitual ways, and prevent reflection regarding what parents most want for their children and how that translates into what they do every day. Further, it is likely that many UWEC parents are not aware, yet would change accordingly if they knew that there are parenting practices associated with children growing to help others with initiative in complex ways, and that participating in household work can be enriching for children (e.g., Rogoff, 2014; Coppens et al., 2016).

How parents modify practices regarding child helping as children develop is another important consideration. There is evidence that UWEC parent socialization practices of encouraging and praising differ in their effects according to toddlers’ age (Dahl, 2015; Dahl et al., 2017), suggesting the importance of how socialization practices may change over time. While toddlers show enthusiasm to help across cultures, in many UWEC families, children beyond toddlerhood have presumably lost interest and resist helping; details regarding the role of socialization in children losing interest in helping are needed (Alcalá et al., 2014). Kärtner (2018) describes differences in prosociality across cultures emerging after toddlerhood; consistent parenting differences may start to take effect then, and/or parent practices may change in different ways (see Mosier and Rogoff, 2003). Particular attention should be paid to 15–16 months of age, an age that may be transitional (Dahl, 2015; Dahl et al., 2017), and 2–3 years of age, an age when some UWEC parents described discouraging children from helping, did not believe children were capable of helping effectively or spontaneously, or described children’s helping with household work as play and/or as events curated by parents (Coppens and Rogoff, 2017; Coppens et al., 2020). At 2–3 years of age, children gain strength yet still lack understanding of household tasks; some UWEC parents may start to divert children’s interests from helping when bids to help are potentially more disruptive and require more guidance, especially if parents do not regard children’s helping with initiative as possible. Evidence is needed to test this theory.

Whiting and Whiting’s (1973) call for change remains relevant. Future research should explore the possibility for change. Unrealized parent values may provide motivation to relieve tension between UWEC parent practices and values (Lönnqvist et al., 2006).

## How Suggested Questions Could Be Answered

Investigating a conflict between UWEC parenting practices and values regarding children’s helping calls for in-home observations for a rich understanding of what happens every day, and deeper understanding of household/family structural factors integral to daily practices. Mixed methods or qualitative studies should probe parent phenomenological perspectives, beliefs, and motivations to allow for theory-building.

Longitudinal studies should track parent practices and beliefs through important periods of child development, from the point in development children are first observed to help, approximately 12 months of age (Liszkowski et al., 2008; Dahl, 2015), to past age 3, as suggested above. Longitudinal studies, cross-cultural or otherwise, are also lacking, despite well-established relationships between different communities’ early parenting practices and beliefs, and children’s development of helping (Rogoff et al., 1993; de Haan, 1999; Gaskins, 1999; Coppens and Rogoff, 2017).

Future UWEC parent interviews, observations and experiments should explore the relative importance of parents’ values, what reasons they might have for their priorities, and what daily practices may relate to their values ranking. Other values and situational factors should be explored to see if they limit parents’ ability to use practices that are associated with children helping extensively with initiative for the benefit of the group, and researchers can ask whether parents know about those practices and related child helping. Exploring differences in how parenting practices/beliefs change over time across cultures would help these pursuits considerably, through direct longitudinal comparison.

Interventions hold promise to test the malleability of UWEC socialization practices, and develop methods for change. Parents could learn to attend to toddlers’ cues through research indicating toddlers’ helping motivations and capabilities/potential, and learn practices that go along with children’s extensive initiative to help others and the group.

## Discussion

Evidence of cultural variation should be integrated into all stages of developmental research; cross-cultural study and describing universal patterns of development are not theoretically separate. Studies and theory exploring universal patterns still often consider only UWEC patterns, inaccurately perpetuating the bias that UWEC patterns and practices are “mainstream” (e.g., Arnett, 2008; Henrich et al., 2010; Nielsen et al., 2017; Alcalá et al., 2018; Morelli et al., 2018). Developmental education programs have not corrected this bias, despite the feasibility of revising curricula (see Keller, 2018). The knowledge upon which scholars build research expertise must be accurate to enable real change.

Studies regarding UWEC socialization of helping can improve coherence with cross-cultural studies. Elements pointed out in the extensive work mentioned here could be incorporated, including: children’s access to participation in household work; parents encouraging autonomy vs. controlled participation; PSE and parental sensitivity; parents’ perception of toddlers’ motivations and abilities behind bids to help; child-initiated helping; parents redirecting or not supporting bids to help; toddler interactions with siblings; parent/child focus on group goals and benefit; children’s situational awareness; and how parents regard daily tasks and parents’/children’s related roles.

An example of continued bias is the rarity of research exploring universal benefit of practices from non-UWEC communities (cf. Rogoff et al., 2017; Rosado-May et al., 2020), while many researchers assume UWEC practices have universal benefit (see Arnett, 2008; Morelli et al., 2018). Overcoming this bias may allow the myriad of extant patterns and practices to benefit many communities, and global society (Rogoff et al., 2017; Rosado-May et al., 2020).

The variety of studies suggested here calls on researchers to assume a non-hierarchical view of diverse, cross-cultural ways of doing and being (Rogoff, 2003), and of researchers’ ways of discovering knowledge. Academic divides exist partly because there are differences in beliefs about what knowledge is and how to get it. The question most important for researchers to answer nowadays may be how to achieve true epistemic diversity. Research may best be understood as finding useful ways to describe an infinitely complex reality. This complexity is clear in cross-cultural studies, and is most productively acknowledged and fully embraced. Increasing efforts to communicate and coordinate across research groups in the realm of the socialization of helping and UWEC conflicts between values and practice is a feasible step, and could have global implications regarding what is discovered and how.

## Author Contributions

LF is the sole author, having conceived, written, and revised this manuscript.

## Conflict of Interest

The author declares that the research was conducted in the absence of any commercial or financial relationships that could be construed as a potential conflict of interest.

## Publisher’s Note

All claims expressed in this article are solely those of the authors and do not necessarily represent those of their affiliated organizations, or those of the publisher, the editors and the reviewers. Any product that may be evaluated in this article, or claim that may be made by its manufacturer, is not guaranteed or endorsed by the publisher.
